# Supplementary material for: Simulation of spontaneous G protein activation reveals a new intermediate driving GDP unbinding
Source: eLife. 2018 Oct 5;7:e38465. doi: 10.7554/eLife.38465 (PMC6224197; doi:10.7554/eLife.38465)
Supplement: Figure 3—source data 1. [file elife-38465-fig3-data1.docx]

**Figure 3A – source data.** Measurements comparing tilting and translation of H5 across PDB structures and MD simulation.

| **Construct description** | **PDB ID** | **H5 Tilting Distance (Å)** | **H5 Vertical translation distance (Å)** | **H5 Tilting residues used** | **H5 Translation residues used** |
| --- | --- | --- | --- | --- | --- |
| Gαq-GDP | 3AH8 | 13.5 | 10.6 | Tyr325 to Leu349 | Thr334 to Phe341 |
| Gαq after rate limiting step from MD | N/A | 15.1 | 11.1 | Tyr325 to Leu349 | Thr334 to Phe341 |
| Gαi-GDP | 1GP2 | 10.3 | 10.2 | Tyr320 to Ile343 | Thr329 to Phe336 |
| Gαi-µOR | 6DDF | 14.6 | 13.0 | Tyr320 to Ile343 | Thr329 to Phe336 |
| Gαi-A1AR | 6D9H | 13.8 | 10.1 | Tyr321 to Ile344 | Thr330 to Phe327 |
| Gαi-Rhodopsin | 6CMO | 15.8 | 10.7 | Tyr320 to Ile343 | Thr329 to Phe336 |
| Gαo-5HT1B | 6G79 | 13.1 | 14.2 | Tyr310 to Ile333 | Thr319 to Phe326 |
| Gαs-B2AR | 3SN6 | 12.8 | 14.6 | Tyr360 to Ile383 | Thr369 to Phe376 |
